# Current News and Opinion

**Published:** 1883-12

**Authors:** 


### Current News and Opinion.

---

### DR. GREGG VERSUS BACTERIA.

Different scientific and non-scientific journals have, for some time, been making allusions to the researches of R. R. Gregg, M. D., of Buffalo, who thinks he has made discoveries that entirely disprove the active influence of bacteria in the promotion of putrefaction, and quite drive a consideration of them from the domain of medicine. He lately read a labored paper, entitled "The Bacterian or Germ Theory of Disease Overturned," before the Buffalo Microscopical Society. That his assertions might be thoroughly considered, the society appointed a committee of acknowledged microscopical and scientific ability, to examine his methods and analyze his theories. That committee has made an exhaustive report, of which we present an abstract.

Dr. Gregg, in his paper, said: "I have controverted the theory of the bacterists for years, and contended that all their so-called bacteria of disease were nothing more or less than so many different forms of fibrine."

After detailing Dr. Gregg's method of conducting his examination, the objective used, and the very crude methods of illumination, the committee says:—

"It will be seen that the doctor disregards all methods of identification save one, namely: appearances in a dry film of albuminous material, seen under circumstances about as conclusive as that of taking the topography of a country from a balloon through a fog. * * * * The specimens prepared from rotting fibrine were made up of bacteria, as is plainly revealed by careful staining and micro-chemical tests. The committee have separately and collectively made experiments to test the identity of fibrinous forms with bacteria, and it may be positively stated that the results are in every case negative. In no single instance was fibrine found to imitate bacteria in its behavior toward the various reagents and chemical tests that are used in the study of bacterian forms. * * * * It is unnecessary to follow this subject further. It must be apparent to any one, having one grain of manipulative skill and the slightest acquaintance with correct methods of microscopical investigation, that no reliance can be placed upon such observations, and that all conclusions based upon them may be dismissed without serious consideration."

This report should finally dispose of and put at rest the theories of Dr. Gregg concerning the spontaneous organization of fibrine, and its simulation of bacterian forms.

### ORAL PATHOLOGY.

A red line on the gums, with fetor and metallic taste, indicates ptyalism.

A blue line—lead poisoning.

Great sponginess, with sloughing and great fetor—scurvy.

A red line about the teeth and along the gums—periostitis.

Purple gums and purulent discharge—necrosis.

Gums hot, red, swollen, very tense—phlegmon.

Gums inflamed and soft, with fluctuation—alveolar abscess.

Swollen gums, fetid discharge, mucus patches, shallow ulcers under the tongue, eroded palate, eruption of mouth, skin, and scalp, gums everted, fetid matter from necks of teeth—syphilis.

A white-coated tongue denotes—febrile disturbance.

A brown, moist tongue—indigestion.

A brown, dry tongue—depression, blood-poisoning, typhoid fever.

A red, moist tongue—feebleness, exhaustion.

A red, dry tongue—inflammatory fever.

A red, glazed tongue—general fever, loss of digestion.

A tremulous, moist and flabby tongue—feebleness, nervousness.

A glazed tongue, with blue appearance—tertiary syphilis.

---

### AMERICAN ACADEMY OF DENTAL SCIENCE.

The regular annual meeting of the American Academy of Dental Science, was held on the 7th of November, 1883, at Young's Hotel, in Boston. The annual address was delivered by Dr. N. W. Kingsley, of New York. The dinner, which is a prominent feature of the annual gathering of the Academicians, was as usual, a very social and pleasant affair. The officers elected for the ensuing year are as follows:

George T. Moffatt, President ; J. H. Batchelder, Vice-President ; T. E. Banfield, Recording Secretary; E. B. Hitchcock, Corresponding Secretary; E. H. Smith, Treasurer; H. C. Meriam, Librarian; C. P. Wilson, E. C. Briggs, J. S. Mason, Executive Committee. Drs. T. O. Loveland and A. B. Jewell, were elected Active Fellows.

The Treasurer's report showed a flourishing financial condition.

H. F. HAMILTON, Secretary.

### DAMAGES FOR ACCIDENTS DURING ANÆSTHESIA.

A suit brought against a dentist in New York city for negligence in allowing a piece of a tooth to drop into a patient's throat, which after causing the patient to suffer with a cough was expectorated at the end of a month, was recently decided against the defendant, who was obliged to pay five hundred dollars damages. The case was appealed but the judgment was sustained. This recalls the case of a boy who died under the influence of nitrous-oxide gas, being suffocated by a molar tooth that slipped out of the forceps and became impacted in the larynx. In such cases a dentist should be both competent and willing to perform tracheotomy, for the patient will generally die of suffocation before a surgeon can be summoned.—*Phil. Med. Times.*

---

### DR. LORD'S INSTRUMENTS.

No dentist should be without Dr. Lord's sickle-shaped instruments. For smoothing approximate surfaces of incisors and bicuspids when stained or menaced by decay, they do excellent service. They are also valuable for trimming ragged marginal walls of approximal cavities, and for finishing fillings in such cavities, whether gold or plastic. A familiarity with these instruments suggests their use in many cases. For removing salivary calculus, they render efficient aid; also for tucking the rubber dam about the cervical portion of such teeth as it surrounds, preparatory to filling.

C. E. F.

---

### AN IRRESPONSIBLE CALL.

Some of the English journals are publishing a circular issued from New York, inviting the co-operation of foreign dentists in the formation of an International Progressive Science Society, and they indulge in covert allusions to the assurance that prompts the movement. Before they bring a charge of assumption against American dentists generally, they should ascertain that the circular in question was issued with the knowledge and approbation of the profession here, or that it comes from a source that will command the attention of our dentists.

A RIGHTEOUS JUDGMENT.

Some conscienceless parties having placed upon the market an imitation of Horsford's Acid Phosphate, the Supreme Court of Rhode Island, in July last, enjoined its further sale in any package which was a substantial or colorable imitation of Horsfords' preparation. This injunction was violated, and the Court on the 24th of September issued a writ of attachment against the parties, and fined them three hundred dollars each. So much for trying to obtain a reputation by stealing it.

---

DIO LEWIS'S MONTHLY.

For a generation Dr. Lewis has been known as an original and pungent writer upon medical and hygienic science, and his crisp paragraphs have been extensively quoted, especially by the secular press. He has now established a monthly journal in New York, which is quite unique in its scope, attractive in its appearance, and very readable as a matter of course. Each number contains about one hundred pages, and there is not a dull paragraph in a volume.

---

LYON'S TOOTH-POWDER.

The profession is familiar with Dr. I. W. Lyon's Tooth Tablets, as they have been a standard dentifrice for years. The tooth powder that he has recently placed upon the market is equally good, less in price, and is put up in enameled tin bottles, with a sprinkler top, a form that will at once commend itself as one not liable to the mishaps of the glass bottle.

O. E. H.

---

TO REMOVE WARTS.

These troublesome excrescences may be removed by frequently touching them with chromic acid. Glacial acetic acid will produce the same result, but not as quickly.—*Scientific American.*

